# Evidence linking oxidative stress, mitochondrial dysfunction, and inflammation in the brain of individuals with autism

**DOI:** 10.3389/fphys.2014.00150

**Published:** 2014-04-22

**Authors:** Daniel A. Rossignol, Richard E. Frye

**Affiliations:** ^1^Rossignol Medical CenterIrvine, CA, USA; ^2^Department of Pediatrics, Arkansas Children's Hospital Research Institute, University of Arkansas for Medical SciencesLittle Rock, AR, USA

**Keywords:** autism, brain, oxidative stress, mitochondrial dysfunction, inflammation

## Abstract

Autism spectrum disorders (ASDs) are a heterogeneous group of neurodevelopmental disorders that are defined solely on the basis of behavioral observations. Therefore, ASD has traditionally been framed as a behavioral disorder. However, evidence is accumulating that ASD is characterized by certain physiological abnormalities, including oxidative stress, mitochondrial dysfunction and immune dysregulation/inflammation. While these abnormalities have been reported in studies that have examined peripheral biomarkers such as blood and urine, more recent studies have also reported these abnormalities in brain tissue derived from individuals diagnosed with ASD as compared to brain tissue derived from control individuals. A majority of these brain tissue studies have been published since 2010. The brain regions found to contain these physiological abnormalities in individuals with ASD are involved in speech and auditory processing, social behavior, memory, and sensory and motor coordination. This manuscript examines the evidence linking oxidative stress, mitochondrial dysfunction and immune dysregulation/inflammation in the brain of ASD individuals, suggesting that ASD has a clear biological basis with features of known medical disorders. This understanding may lead to new testing and treatment strategies in individuals with ASD.

## Introduction

Autism spectrum disorders (ASD) are a group of neurodevelopmental disorders that are defined by behavioral observations including communication and social interaction problems and repetitive behaviors (APA, 1994). ASD affects an estimated 1 out of 88 individuals in the United States (U.S.) (Baio, 2012) with four times more males than females being affected (Rice, 2007). The etiology of ASD is unclear at this time. Although several genetic syndromes, including Fragile X and Rett syndrome, have been associated with ASD; genetic defects account for only a small percentage of ASD cases (Schaefer et al., 2013).

Although many of the cognitive and behavioral features of ASD are thought to arise from dysfunction of the brain, evidence from many fields of medicine has documented physiological abnormalities in organs besides the brain that are associated with ASD, suggesting that, in some individuals, ASD arises from systemic, rather than organ specific abnormalities (Rossignol and Frye, 2012a). Specifically, in recent decades, research and clinical studies have implicated physiological and metabolic systems that transcend specific organ dysfunction, such as immune dysregulation and inflammation, abnormalities in redox regulation and oxidative stress, and dysfunction of energy generation and mitochondrial systems (Ming et al., 2008; Rossignol and Frye, 2012a). In this context, ASD may arise from, or at least involve, systemic physiological abnormalities rather than being a purely central nervous system (CNS) disorder (Herbert, 2005), at least in a subset of individuals with ASD. However, because the CNS is affected in ASD, examining physiological abnormalities in the brain may reveal more about what is abnormal than inspecting abnormalities in blood or urine samples.

A number of studies have reported evidence of oxidative stress in individuals with ASD (Yorbik et al., 2002; James et al., 2004, 2006, 2009a,b; Ming et al., 2005; Chauhan and Chauhan, 2006; Yao et al., 2006; Al-Gadani et al., 2009; Melnyk et al., 2012; Rose et al., 2012a; Rossignol and Frye, 2012a) and their parents (James et al., 2008). Genetic variations in glutathione-related pathways have been observed in ASD (Boris et al., 2004; James et al., 2006; Bowers et al., 2011; Frustaci et al., 2012) and have been correlated in some studies with ASD behaviors (Goin-Kochel et al., 2009; Guo et al., 2012). Several case-control studies have reported lower concentrations of reduced glutathione (GSH), higher levels of oxidized glutathione (GSSG) and a decrease in the GSH/GSSG redox ratio (James et al., 2004, 2006, 2009b), along with a lower mitochondrial GSH reserve (James et al., 2009a) in individuals with ASD compared to controls. In addition, in some studies, lower GSH levels (Adams et al., 2009) and markers of increased oxidative stress (Ghezzo et al., 2013) have been correlated with ASD severity. Markers of oxidative stress have also been correlated with the severity of gastrointestinal problems in ASD individuals (Gorrindo et al., 2013). Notably, these aforementioned studies examined peripheral markers of oxidative stress, including those found in blood and urine. Recently, a number of studies have reported evidence of oxidative stress in post-mortem brain samples from individuals with ASD compared to controls (Evans et al., 2008; López-Hurtado and Prieto, 2008; Sajdel-Sulkowska et al., 2008, 2009; Palmieri et al., 2010; Chauhan et al., 2011; Sajdel-Sulkowska et al., 2011; Chauhan et al., 2012a; Rose et al., 2012b; Gu et al., 2013a,b; Tang et al., 2013).

Multiple studies have also reported evidence of mitochondrial dysfunction in individuals with ASD (Rossignol and Bradstreet, 2008; Weissman et al., 2008; Giulivi et al., 2010; Guevara-Campos et al., 2010; Shoffner et al., 2010; Zhang et al., 2010; Dhillon et al., 2011; Frye and Rossignol, 2011; Chauhan et al., 2012b; Frye, 2012; Frye and Rossignol, 2012a; Rossignol and Frye, 2012a,b; Frye et al., 2013a,b; Frye and Rossignol, 2013). In some studies, biomarkers of mitochondrial dysfunction have been associated with autistic behaviors or autism severity (Minshew et al., 1993; Mostafa et al., 2005). One systematic review reported that over 30% of children with ASD have biomarkers of abnormal mitochondrial function suggesting that a relatively high percentage of individuals with ASD might have some degree of mitochondrial dysfunction (Rossignol and Frye, 2012b). Another study reported that up to 50% of children with ASD have biomarkers of mitochondrial dysfunction that are valid (that is, they correlate with other biomarkers of mitochondrial dysfunction) and are consistently abnormal (that is, they are repeatedly abnormal) (Frye, 2012). However, like the studies on oxidative stress and ASD, most of the published literature concerning mitochondrial dysfunction has examined blood and urine samples. A number of studies recently have reported evidence of mitochondrial dysfunction in ASD brain samples compared to controls (Palmieri et al., 2010; Chauhan et al., 2011; Anitha et al., 2012, 2013; Ginsberg et al., 2012; Rose et al., 2012b; Tang et al., 2013).

Finally, a number of studies have reported evidence of immune dysregulation and/or inflammation in individuals with ASD (Gupta et al., 2010; Onore et al., 2012; Rossignol and Frye, 2012a; Depino, 2013; Gesundheit et al., 2013; Goines and Ashwood, 2013), including gene changes pertaining to the immune system (Michel et al., 2012; Poultney et al., 2013). In some studies, biomarkers of inflammation or immune dysregulation have been correlated with ASD severity (Mostafa and Kitchener, 2009; Al-Ayadhi and Mostafa, 2011, 2012, 2013; Khakzad et al., 2012; Mostafa and Al-Ayadhi, 2012) and an elevation in TNF-alpha has been reported in ASD lymphocytes (Malik et al., 2011a) and in amniotic fluid in children who develop autism (Abdallah et al., 2013). Particular interest surrounds elevations found in autoantibodies to brain elements and other important molecular targets such as the folate receptor autoantibody (Connolly et al., 1999; Rossignol and Frye, 2012a; Frye et al., 2013c). Although there have been a large number of studies examining immune abnormalities in ASD, almost all of these studies have examined blood and urine samples. However, some studies have recently reported evidence of brain-related immune dysregulation or inflammation in ASD compared to controls (Vargas et al., 2005; Chez et al., 2007; Garbett et al., 2008; Li et al., 2009; Morgan et al., 2010; Wei et al., 2011; Young et al., 2011; Rose et al., 2012b; Suzuki et al., 2013).

Recently, an interrelationship between oxidative stress, mitochondrial dysfunction, and/or inflammation has been reported in some individuals with autism (James et al., 2009a; Mostafa et al., 2010; Zhang et al., 2010; Rose et al., 2012b; Frye et al., 2013a; Napoli et al., 2013; Theoharides et al., 2013). In this manuscript, we concentrate on studies that have documented these physiological abnormalities specifically in the CNS of individuals with ASD. Reviewing the evidence for these physiological abnormalities specifically in the CNS is important for several reasons. Firstly, the CNS is protected from the rest of the body by the blood-brain barrier. Although there is evidence that these physiological abnormalities are present in non-CNS tissue in individuals with ASD, it does not necessarily mean that they are present in the CNS. Demonstrating that these abnormalities also affect the brain would suggest that brain dysfunction in individuals with ASD is not necessarily only secondary to systematic abnormalities, but that the same abnormalities that influence peripheral organs also directly influence brain function. Secondly, there are particular patterns of abnormalities in the CNS that are associated with ASD. Indeed, abnormalities in ASD have been reported in the frontal and temporal cortices, the hippocampus and amygdala as well as the cerebellum. Determining whether these physiological abnormalities are also present in these brain areas would provide insight into whether they could be involved in the pathological mechanisms that result in ASD. Thus, this manuscript reviews the evidence for oxidative stress, mitochondrial dysfunction and immune dysregulation/inflammation in the brains of individuals with ASD compared to controls as well as the evidence linking these abnormalities.

## Studies of oxidative stress in the ASD brain

A number of studies have reported evidence of oxidative stress in post-mortem brain samples obtained from individuals with ASD compared to controls (Table 1). These studies have demonstrated a decrease in GSH, the major cellular antioxidant, oxidative damage to proteins, lipids and deoxyribonucleic acid (DNA) as well as alternations in the activity of enzymes important in redox metabolism.

**Table 1:** **Studies of oxidative stress in the ASD brain**.

**Year published**	**Citation**	**No. of ASD**	**No. of controls**	**Major findings**
2008	Evans et al., 2008	5	5	Increase in 3 oxidative damage markers in cerebellum, hippocampus and BA39 in ASD group
2008	López-Hurtado and Prieto, 2008	8	7	Increase in lipofuscin containing cells in BA22, BA44, and BA39 in ASD group
2008	Sajdel-Sulkowska et al., 2008	9	10	Significant elevation in 3NT in ASD group; 3NT correlated with cerebellar mercury concentration
2009	Sajdel-Sulkowska et al., 2009	8	7	3NT correlated with neurotrophin-3 in the ASD brain; 8-hydroxydeoxyguanosine higher in cerebellum in ASD group
2010	Palmieri et al., 2010	6	6	Three-fold higher oxidative damage to mitochondrial proteins in BA41/42 or BA22 in ASD group
2011	Chauhan et al., 2011	8	8	Increased lipid hydroperoxides in temporal lobes and cerebellum in ASD group
2011	Sajdel-Sulkowska et al., 2011	2	2	Elevated 3NT in orbitofrontal cortex, Wernicke's area, cerebellar vermis and pons in ASD group
2012	Chauhan et al., 2012a	10	10	GSH/GSSG and reduced GSH levels both significantly lower in cerebellum and temporal lobes in ASD group
2012	Rose et al., 2012b	15	15	GSH/GSSG and GSH both significantly lower in cerebellum and BA22 in ASD group; 3NT elevated in cerebellum and BA22 in ASD group; 8-oxo-deoxyguanosine elevated in cerebellum and BA22 in ASD group and correlated with GSH/GSSG
2013	Gu et al., 2013b	10	10	Glutathione peroxidase, Glutathione-S-transferase, glutamate cysteine ligase decreased in cerebellum in ASD group
2013	Muratore et al., 2013	10	10	Similar 8-hydroxyguanosine in the frontal cortex (BA 9, 22, 41, 42, or 46) between groups. Significantly lower methionine synthase mRNA along with lower levels of homocysteine and cystathionine in these same areas in the autism brains compared to controls, suggestive of adaptive responses to oxidative stress
2013	Tang et al., 2013	20	25	Diminished superoxide dismutase 2 activity in BA21 in ASD group; 8-oxo-deoxyguanosine higher in temporal lobe in ASD group

Several studies have reported GSH abnormalities in the brain tissue of individuals with ASD. In one study of 10 individuals with autism and 10 age-matched controls, GSH/GSSG and reduced GSH levels were both significantly lower in the cerebellum and temporal cortex in the autism group compared to controls (Chauhan et al., 2012a). Another study of 15 individuals with autism and 15 controls reported significantly lower GSH and GSH/GSSG levels in the cerebellum and Brodmann area 22 (BA22) in the autism group. Interestingly, these markers of GSH metabolism did not correlate with age, suggesting that the oxidative stress observed was a chronic condition (Rose et al., 2012b).

Enzymes important for redox metabolism have also been found to be altered in brain tissue derived from individuals with autism. In a study of temporal lobe brain samples (BA21) taken from 20 individuals with autism and 25 controls, Tang et al. observed a decrease in superoxide dismutase 2 activity in brain samples from the autism group (Tang et al., 2013). Activities of glutathione peroxidase, glutathione-S-transferase, and glutamate cysteine ligase were each significantly decreased in the cerebellum of the brains of 10 individuals with autism compared to 10 controls as reported by Gu et al. (2013b). Finally, significantly lower methionine synthase mRNA along with lower levels of homocysteine and cystathionine were observed in the frontal cortex (BA 9, 22, 41, 42, or 46) in the brains of 10 individuals with autism (ages 4–30 years) compared to 10 age- and sex-matched controls, suggestive of adaptive responses to oxidative stress (Muratore et al., 2013).

Some studies have reported oxidative damage to brain lipids in ASD. One of the first studies reported a significant increase in lipofuscin containing cells, a marker of oxidative stress, in 3 language areas of the brain (BA 22, 39, and 44) in 8 males with autism compared to 7 male controls (López-Hurtado and Prieto, 2008). Another study reported significant immunoreactivity to 3 markers of oxidative damage (carboxyethyl pyrrole (CEP) and iso[4]levuglandin (iso[4]LG)E2-protein adducts as well as heme oxygenase-1) in cerebellar, hippocampal, and BA39 brain samples from 5 subjects with autism but not in any of 5 controls (Evans et al., 2008). Finally, one study of 8 children with autism and 8 controls reported significantly higher levels of lipid hydroperoxides (an oxidative stress marker) in the temporal cortex and cerebellum in the autism group; mitochondrial dysfunction was also observed in this study in the same areas (Chauhan et al., 2011).

Other studies have documented significant oxidation of proteins in brain tissue by examining levels of 3-nitrotyrosine (3NT). One study which examined brain tissue from 9 individuals with autism and 10 controls reported that 3NT in the cerebellum was significantly elevated in the autism group and was significantly correlated (*r* = 0.80) with cerebellar mercury levels (Sajdel-Sulkowska et al., 2008). In a follow-up study, these investigators found that neurotrophin-3, a neurotrophin critical for normal brain growth and differentiation, was positively correlated (*r* = 0.83) with 3NT in the cerebellum samples from 8 individuals with ASD compared to 7 controls. The investigators suggested that increased neurotrophin-3 could affect brain development and growth and lead to cerebellar overgrowth, and that this increase in neurotrophin-3 could be due to increased levels of oxidative stress in the developing brain (Sajdel-Sulkowska et al., 2009). The same group examined 2 children with autism and 2 age-matched controls and reported that 3NT varied widely in the autism brain samples compared to a uniformly low level in the control brains; the elevated levels of 3NT in the two autism cases were found in areas of the brain responsible for sensory and motor coordination, speech processing, memory and social behavior (orbitofrontal cortex, Wernicke's area, cerebellar vermis and pons) (Sajdel-Sulkowska et al., 2011). A recent study of 15 individuals with autism and 15 controls reported a significant elevation of 3NT in the cerebellum and BA22 in the autism group (Rose et al., 2012b). Finally, one study of 6 individuals with non-syndromic autism and 6 controls examined brain tissue from the superior temporal gyrus (BA 41/42 or 22) and reported a 3-fold higher level of oxidative damage to mitochondrial proteins in the autism group compared to controls (Palmieri et al., 2010).

Other studies have documented increased oxidation of DNA in the brain tissue of individuals with ASD. In one study of 20 individuals with autism and 25 controls, 8-oxo-deoxyguanosine was elevated in the temporal lobe of the autism group (Tang et al., 2013). In another study of 8 individuals with autism and 7 controls, the levels of 8-hydroxydeoxyguanosine were 63% higher in the cerebellum of the autism group (*p* = 0.23) (Sajdel-Sulkowska et al., 2009). Another study of 15 individuals with autism and 15 controls reported significant elevations in 8-oxo-deoxyguanosine in the cerebellum and BA22 (superior temporal lobe) in the autism group. In this study, DNA damage was found to significantly correlate with the GSH/GSSG redox ratio (Rose et al., 2012b). However, a study of 10 individuals with autism (ages 4–30 years) and 10 age- and sex-matched controls reported similar 8-hydroxyguanosine, a marker of oxidative damage to RNA, in the frontal cortex (BA 9, 22, 41, 42, or 46) (Muratore et al., 2013).

Overall, as reviewed above, there are many lines of evidence supporting the notion of increased levels of oxidative stress in key regions of the brain in individuals with ASD. First, oxidative damage to many cellular elements has been reported. Three studies have reported biomarkers of oxidative damage to lipids in five brain areas: temporal (López-Hurtado and Prieto, 2008; Chauhan et al., 2011), parietal (Evans et al., 2008; López-Hurtado and Prieto, 2008), frontal lobes (López-Hurtado and Prieto, 2008), hippocampus (Evans et al., 2008), and cerebellum (Evans et al., 2008; Chauhan et al., 2011). Biomarkers of oxidative damage to proteins have been reported in four brain areas in 4 studies: the cerebellum (Sajdel-Sulkowska et al., 2008, 2011; Rose et al., 2012b), frontal (Sajdel-Sulkowska et al., 2011), temporal lobes (Palmieri et al., 2010; Sajdel-Sulkowska et al., 2011; Rose et al., 2012b), and the brainstem (Sajdel-Sulkowska et al., 2011). Biomarkers of oxidative damage to DNA have been reported in two areas of the brain in 3 studies: the temporal lobe (Rose et al., 2012b; Tang et al., 2013) and cerebellum (Sajdel-Sulkowska et al., 2009).

Secondly, biomarkers indicative of increased reactive oxygen species have been reported. Abnormal levels of glutathione (low reduced glutathione, elevated oxidized glutathione and depressed glutathione redox ratio) have been found in two brain areas in 2 studies: the temporal cortex (Chauhan et al., 2012a; Rose et al., 2012b) and cerebellum (Rose et al., 2012b), and in one study a response to oxidative stress (increased heme oxygenase-1) has been reported in the parietal and frontal lobes and the cerebellum (Evans et al., 2008).

Lastly, two studies have demonstrated that certain essential enzymes involved in controlling reactive oxygen species and producing glutathione are reduced in three brain areas, including the temporal lobe (Tang et al., 2013), cerebellum (Tang et al., 2013), and frontal cortex (Muratore et al., 2013).

Despite this converging evidence of oxidative stress in several brain areas in multiple studies, it is clear that there are several limitations to this evidence. First, because of the limited number of brain tissue samples generally available, the majority of studies have only examined a limited number of brain samples (one study only used 2 samples in each group). Thus, although most studies have included a brain sample from the temporal area or cerebellum, specific areas of the brain were not consistently analyzed across studies. However, because many of these studies demonstrate significant effects despite small sample sizes, the effects found are rather robust across ASD subjects and brain regions. This raises the possibility that these processes are pervasive in ASD. Since it is well accepted that the general ASD population is composed of heterogeneous phenotypes, larger samples sizes are needed to determine if subgroups of children with ASD exist who have significant differences in brain redox environments.

Still, there are larger questions that must be answered beyond confirming the notion that oxidative stress is present in the brain of children with ASD. For example, it is possible that the reduced transportation of folate into the brain as a consequence of the folate receptor alpha autoantibody or mitochondrial dysfunction could reduce the function of methylation and glutathione metabolism specifically within the brain leading to some of the findings described above (Frye et al., 2013c). However, many of these same findings reported for the brain (oxidative damage to lipids, protein and DNA, glutathione abnormalities, reduced function of enzymes essential for regulating oxidative stress) have been found in the blood, immune cells and cell lines derived from individuals with ASD, thereby raising the question of whether these findings are specific for the brain or whether they represent a more general process (Frye et al., 2013c; Frye and James, 2014). Lastly, the etiology of these abnormalities is not clear, as both increases in pro-oxidant influences and reductions in antioxidant defenses have both been associated with ASD (Chauhan and Chauhan, 2006) and it is clear that there are no simple genetic abnormalities that account for these findings (Frustaci et al., 2012; Frye and James, 2014).

## Studies of mitochondrial dysfunction in the ASD brain

Evidence of mitochondrial dysfunction in the brain of individuals with autism has been reported by using magnetic resonance imaging techniques. Other studies have examined postmortem brain samples from individuals with ASD compared to controls. Such studies have demonstrated decreases in electron transport chain (ETC) complex and tricarboxylic acid (TCA) cycle enzyme activities, as well as differences in mitochondrial gene expression in the brain tissue of individuals with autism compared to controls (Table 2).

**Table 2:** **Studies of mitochondrial dysfunction in the ASD brain**.

**Year published**	**Citation**	**No. of ASD**	**No. of controls**	**Major findings**
1993	Minshew et al., 1993	11	11	Markers of mitochondrial function were abnormal in ASD brain and correlated with ASD behaviors
1999	Chugani et al., 1999	9	5	Elevated lactate in the frontal lobe of 1 out of 9 children with ASD; NAA was reduced in the cerebellum of the ASD group
2003	Friedman et al., 2003	45	28	No significant difference in mean lactate in ASD and control groups
2010	Palmieri et al., 2010	6	6	Higher level of oxidative damage to mitochondrial proteins in BA41/42 or BA22 in ASD group; complex IV activity higher in ASD group
2011	Chauhan et al., 2011	8	8	Lower ETC complex activities in cerebellum, frontal lobe and temporal lobe in ASD group
2012	Anitha et al., 2012	8	10	Decreased expression of mitochondrial genes in anterior cingulate gyrus, motor cortex and thalamus in ASD group
2012	Corrigan et al., 2012	54	76	No significant difference in mean lactate in ASD and control groups
2012	Ginsberg et al., 2012	9	9	Decreased ETC complex gene expression in cerebellum and BA19 in ASD group
2012	Rose et al., 2012b	15	15	Significantly decreased aconitase activity in cerebellum and BA22 in ASD group
2013	Anitha et al., 2013	8	10	Reduced expression of ETC genes (complexes I, III, IV, and V) in the anterior cingulate gyrus, thalamus, and motor cortex in ASD group
2103	Golomb et al., 2014	6	6	Brain phosphocreatine reduced in muscle and frontal cortex in children with mitochondrial dysfunction and ASD
2013	Gu et al., 2013a	14	12	Reduced ETC complex I and V activities in frontal lobe in ASD group; a higher mtDNA copy number compared to nuclear DNA in 3 different mitochondrial genes found in ASD group
2013	Tang et al., 2013	20	25	Decreased ETC complex I and IV activities in BA21 in ASD group; higher levels of mitochondrial fission proteins and lower levels of mitochondrial fusion proteins in BA21 of ASD group

The first study to report evidence of mitochondrial dysfunction in the brain in individuals with ASD was a study of 11 ASD individuals and 11 typically developing (TD) controls which reported abnormal levels of brain markers of mitochondrial function in the dorsal prefrontal cortex measured by Phosphorus-31 magnetic resonance spectroscopy (MRS) (including phosphocreatine, αATP, α-adenosine diphosphate, dinucleotides and diphosphosugars) that significantly correlated with the severity of language and neuropsychological deficits in the ASD group but not in the control group (Minshew et al., 1993). Recent studies have demonstrated that Phosphorus-31 MRS is sensitive in detecting metabolic disturbances in children with mitochondrial dysfunction and ASD when muscle and/or brain is examined (Golomb et al., 2014). Most MRS studies have not used Phosphorus-31 imaging but have concentrated on the more popular proton MRS (1H-MRS) technique. The results of 1H-MRS studies are variable in ASD. However, consistent results have been reported for the metabolite N-acetyl-aspartate (NAA). Indeed, studies have consistently demonstrated a decrease in NAA in global white and gray matter of the brain in children with ASD, and in the gray matter of the parietal cortex, the cerebellum, and the anterior cingulate cortex in both children and adults with ASD (Ipser et al., 2012). Other investigators have recently suggested that some of these age-specific changes in gray matter NAA may be different in ASD children compared to TD and developmentally delayed children (Corrigan et al., 2013). NAA is a particularly important brain metabolite as it is not only a marker of neuronal integrity, but a marker of mitochondrial dysfunction (Clark, 1998) since it is exclusively synthesized by the mitochondria of neurons. One study used MRS to measure NAA and lactate in 9 children with ASD and 5 control siblings in the frontal, temporal and cerebellar areas. An elevation in lactate was found in the frontal lobe of one of the 9 children (11%) with ASD, and NAA was reduced in the cerebellum of the ASD group as compared to the control group (Chugani et al., 1999). Another study of 45 children with ASD, 15 children with developmental delay and 13 TD children reported reduced NAA concentrations in the ASD group compared to the TD controls, but no significant difference in mean lactate levels (Friedman et al., 2003). A more recent study of 54 children with ASD, 22 children with developmental delay and 54 TD children found no significant difference in mean lactate levels between groups using 1H-MRS (Corrigan et al., 2012). Although elevations in lactate have been investigated using 1H-MRS in children with ASD, it is likely that the lack of findings in this population is due to the poor sensitivity of 1H-MRS for identifying lactate elevations in the brain (Rossignol and Frye, 2012c).

Several studies have examined ETC function in the brain of children with ASD. One study examined 8 children with autism and 8 controls (4–10 years of age) and reported significantly lower ETC complex activities in the cerebellum, frontal cortex and temporal cortex of the autism group (Chauhan et al., 2011). In another study of temporal lobe brain samples (BA 21) taken from 20 individuals with autism and 25 controls, there were decreased ETC complex I and IV activities and protein content in the ASD group (Tang et al., 2013). In one study of 15 individuals with ASD and 15 controls, the mean activity of the TCA cycle enzyme aconitase was significantly decreased in the cerebellum and temporal lobe (BA22) in the autism group (Rose et al., 2012b). Finally, another study of 14 individuals with autism and 12 controls reported mean reductions in ETC complexes I (31%) and V (36%) activities as well as in pyruvate dehydrogenase (35%) in the frontal cortex in the autism group. This study also reported a higher mitochondrial DNA (mtDNA) copy number compared to nuclear DNA in 3 different mitochondrial genes in the autism group; these latter findings might be related to mitochondrial proliferation (Gu et al., 2013a).

Several studies have examined changes in the expression of mitochondrial genes in brain samples of individuals with autism. Decreased mitochondrial ETC complex gene expression was found in the cerebellum and BA19 (occipital) brain tissue from 9 individuals with autism compared to 9 controls (Ginsberg et al., 2012). In another study, reduced expression of mitochondrial ETC genes, including 11 genes of complex I, five genes of complex III, five genes of complex IV, and seven genes of complex V were reported in the anterior cingulate gyrus, thalamus, and motor cortex derived from 8 patients with autism compared to 10 controls (Anitha et al., 2013). Other studies have examined non-ETC mitochondrial gene expression in brain tissue from individuals with autism. For example, decreased expression of mitochondrial genes, including metaxin 2, neurofilament, SLC25A27, light polypeptide (NEFL) and solute carrier family 25 were found in 8 patients with autism (in the anterior cingulate gyrus, motor cortex and thalamus) compared to 10 controls (Anitha et al., 2012).

One study examined changes in proteins that regulate mitochondrial dynamics. Higher levels of mitochondrial fission proteins and lower levels of mitochondrial fusion proteins were found in temporal lobe brain samples (BA 21) from 20 individuals with autism compared to 25 controls (Tang et al., 2013).

Some studies have implicated an association between mitochondrial dysfunction and oxidative stress in the brain tissue of children with autism by showing that these two problems may coexist in the same brain tissue samples. For example, in temporal lobe brain samples (BA 21), decreased mitochondrial function was found along with increased biomarkers of oxidative damage to DNA and decreased superoxide dismutase 2 activity (Tang et al., 2013). In another study looking at cerebellum, frontal cortex and temporal cortex in 4–10 year olds, brain tissue markers of oxidative stress were observed along with reduced ETC complex activities in the autism group (Chauhan et al., 2011). As previously mentioned, one study of 6 individuals with non-syndromic autism and 6 controls reported a higher level of oxidative damage to mitochondrial proteins in the superior temporal gyrus (BA 41/42 or 22) in the autism group compared to controls. In this study, cytochrome C oxidase (complex IV) activity was also higher in the individuals with autism (Palmieri et al., 2010). Lastly, one study correlated markers of oxidative stress with TCA enzyme function; aconitase activity was inversely correlated with GSH/GSSG in the cerebellum and the temporal lobe (BA 22) in 15 individuals with ASD compared to 15 controls (Rose et al., 2012b).

Overall these studies provide support for mitochondrial dysfunction in the brain of individuals with ASD. MRS studies using both Phosphorus-31 and 1H techniques have examined energy metabolites in the brain of individuals with ASD, although many more studies have used the latter technique. Phosphorus-31 MRS has found abnormal energy metabolites in the frontal cortex (Minshew et al., 1993; Golomb et al., 2014) while 1H-MRS has found a reduction in NAA in the global white and gray matter and the parietal, anterior cingulate and cerebellum areas (Ipser et al., 2012). ETC function has been reported to be depressed in frontal (Chauhan et al., 2011), temporal (Chauhan et al., 2011; Tang et al., 2013), and cerebellar (Chauhan et al., 2011) brain tissue derived from individuals with ASD, with ETC complex I most commonly reported depressed. Other studies noted decreases in the activity of non-ETC mitochondrial enzymes (aconitase, pyruvate dehydrogenase) in frontal (Gu et al., 2013a), temporal (Rose et al., 2012b), and cerebellar (Rose et al., 2012b) tissue derived from children with ASD. Depressed expression of ETC genes in the occipital and cerebellar areas and of ETC and non-ETC genes in the cingulate, thalamus and frontal areas have been reported (Anitha et al., 2013). In addition, changes in genes that control mitochondrial dynamics have been noted in the temporal lobe (Tang et al., 2013).

As with studies on oxidative stress, studies of mitochondrial function in the brain of individuals with ASD are mostly based on small numbers of samples, involve a wide variety of methods, and study various regions of the brain without consistency across studies. Despite these limitations, these studies demonstrate that mitochondrial dysfunction is consistently found in the brain of individuals with ASD. The studies on ETC function are consistent with studies performed on muscle tissue as both demonstrate ETC complex I deficiency as the most prevalent ETC complex abnormality (Rossignol and Frye, 2012b). Several studies have provided powerful evidence for the correspondence between oxidative stress and mitochondrial dysfunction in the same brain samples (Chauhan et al., 2011; Rose et al., 2012b; Tang et al., 2013). This is one step forward to understanding the interaction between oxidative stress and mitochondrial dysfunction. Future studies could make such evidence more powerful by correlating these abnormalities with peripheral markers of mitochondrial dysfunction and oxidative stress as well as examining clinical characteristics.

## Studies of inflammation and immune dysregulation in the ASD brain

Inflammation and immune dysregulation have been observed in both brain tissue and cerebrospinal fluid (CSF) samples from individuals with ASD. In brain tissue, increases in cytokines, expression in immune-related genes, microglial cell activation and other biomarkers of inflammation have also been reported (Table 3).

**Table 3:** **Studies of inflammation/immune dysregulation in the ASD brain**.

**Year published**	**Citation**	**No. of ASD**	**No. of controls**	**Major findings**
2005	Vargas et al., 2005	11	6	Activation of microglia and astroglia in the middle frontal gyrus, anterior cingulate gyrus and cerebellum in ASD group; anti-inflammatory cytokine tumor growth factor–1 and pro-inflammatory macrophage chemoattractant protein-1 were increased in these areas in ASD group; IFN-gamma, MCP-1, TGF-beta2, and IL-8 increased in CSF of ASD group
2005	Zimmerman et al., 2005	12	27	CSF quinolinic acid and neopterin were significantly lower and biopterin was significantly elevated in ASD group
2007	Chez et al., 2007	10	0	Elevated TNF-alpha concentration in CSF compared to serum levels
2008	Garbett et al., 2008	6	6	Increased transcription levels of several immune-related genes in the superior temporal gyrus in ASD group
2008	López-Hurtado and Prieto, 2008	8	7	Reactive gliosis in BA22, BA44, and BA39 in ASD group
2009	Li et al., 2009	8	8	Elevated proinflammatory cytokines in frontal cortex in ASD group
2010	Morgan et al., 2010	13	9	Microglial activation found in dorsolateral prefrontal cortex in ASD group
2011	Malik et al., 2011b	7	7	No significant difference in NF-KappaB expression in the cerebellum and frontal cortex between ASD and controls
2011	Wei et al., 2011	6	6	Elevated IL-6 in cerebellum in ASD group
2011	Young et al., 2011	9	9	NF-KappaB expression in the orbitofrontal cortex was increased in ASD group
2012	Rose et al., 2012b	15	15	Elevated 3-chlorotyrosine level in the cerebellum and temporal cortex in the ASD group
2013	Suzuki et al., 2013	20	20	Microglial activation in multiple brain regions in ASD group

While some studies have reported microglial activation in individuals with autism compared to controls, others have studied differences in the spatial organization of microglial cells in individuals with autism. Microglia are immune cells in the CNS which are activated to eliminate damaged cells or infectious agents through the process of phagocytosis. However, when the microglia are chronically activated, they may increase inflammation through the release of proinflammatory cytokines and free radicals (Dheen et al., 2007). The first study to examine microglia in ASD reported significant activation of microglia and reactive astroglia in the middle frontal gyrus, anterior cingulate gyrus and cerebellum in 11 patients with autism compared to 6 controls (Vargas et al., 2005). The second study examined the dorsolateral prefrontal cortex in 13 males with autism and 9 controls and reported marked microglial activation in 5 out of the 13 autism cases (38.5%) and mild microglial activation in another 4 cases (30.8%) (Morgan et al., 2010). Finally, one study of 20 men with autism and 20 age- and IQ-matched controls reported evidence of microglial activation using positron emission tomography in multiple brain regions (cerebellum, brainstem, corpus callosum, fusiform gyri, superior temporal gyri, anterior cingulate, orbitofrontal, and parietal lobes) in the autism group (Suzuki et al., 2013).

Other studies have noted differences in microglial spatial organization on the microscopic and macroscopic levels in individuals with ASD. One study reported that microglia in the dorsolateral prefrontal cortex were frequently located closer to neurons in 13 individuals with autism compared to 9 controls (Morgan et al., 2012a), while another study reported that microglial cells were associated with reactive astrocytes in 11 patients with autism (Vargas et al., 2005). Another study examined microglia from the fronto-insular and visual cortex in 11 individuals with autism and 12 controls and reported significantly more microglia in the fronto-insular cortex in the autism group; however, this study did not examine microglial activation (Tetreault et al., 2012). Reactive gliosis has been reported in association with microglia activation in one study of 11 patients with autism (Vargas et al., 2005), while another study of 8 males with autism and 7 male controls suggested reactive gliosis along with a greater density of glial cells in 3 different brain areas associated with language (BA 22: speech recognition; BA 44: speech production; and BA 39: reading) in the autism group (López-Hurtado and Prieto, 2008).

Several studies have described increased proinflammatory cytokines in brain tissue from individuals with ASD. One study reported elevations in proinflammatory cytokines (including TNF-alpha, IL-6 and GM-CSF), a Th1 cytokine (IFN-gamma) and a chemokine (IL-8) measured in postmortem frontal cortex brain samples from 8 individuals with autism compared to 8 controls, but no significant differences in Th2 cytokines (IL-4, IL-5, and IL-10) (Li et al., 2009). Another study reported that IL-6 was significantly increased in the cerebellum of 6 children with autism compared to 6 age-matched control children (Wei et al., 2011). In a study of 11 patients with autism, both anti-inflammatory cytokine tumor growth factor-1 and pro-inflammatory macrophage chemoattractant protein-1 were found to be increased in the middle frontal gyrus, anterior cingulate gyrus and cerebellum. Other pro-inflammatory and modulatory cytokines, including interleukin-6 (IL-6), IL-10, MCP-3, eotaxin, eotaxin 2, macrophage-derived chemokine (MDC), chemokine-8 (Ck-8.1), neutrophil activating peptide-2 (NAP-2), monokine induced by interferon (MIG), B-lymphocyte chemoattractant (BLC), leptin and osteoprotegerin, were only consistently elevated in the anterior cingulate gyrus (Vargas et al., 2005).

Other studies have examined the expression of inflammatory genes in brain tissue of individuals with autism. One study reported increased transcription levels of several immune-related genes in the superior temporal gyrus of 6 individuals with autism compared to 6 controls, consistent with neuroimmune activation (Garbett et al., 2008). Two studies examined the NF-KappaB pathway in the brains of individuals with autism. One study reported that NF-KappaB expression in the orbitofrontal cortex was increased in 9 individuals with autism compared to 9 controls (Young et al., 2011). Another study of 7 individuals with autism and 7 controls reported no significant difference in NF-KappaB expression in the cerebellum and frontal cortex (Malik et al., 2011b).

One study of 15 individuals with ASD and 15 controls reported elevated 3-chlorotyrosine levels, a biomarker of chronic inflammation, in the cerebellum and temporal cortex in the ASD group, along with increased markers of oxidative stress and mitochondrial dysfunction (Rose et al., 2012b).

Several studies examined cytokines and other inflammatory markers in the CSF of individuals with ASD. The first study to report findings related to inflammation in the CSF of patients with autism reported significantly elevated IFN-gamma, MCP-1, TGF-beta2, and IL-8 in 6 children with autism compared to 9 child and adult controls (Vargas et al., 2005). One uncontrolled study of 10 children with autism who had a history of regression in language and eye contact examined CSF for inflammatory changes. In this case series, the mean TNF-alpha concentration in CSF was 104.10 pg/mL compared to concurrent serum levels of 2.78 pg/mL (Chez et al., 2007), suggesting production of TNF-alpha in the CNS rather than from systematic inflammation or inflammation of a peripheral tissue. Lastly, one study did not find evidence of immune dysregulation in the CSF in individuals with ASD. In this study of 12 children with autism and 27 controls with other neurological disorders, CSF quinolinic acid and neopterin were significantly lower and biopterin was significantly elevated in the autism group (Zimmerman et al., 2005).

The evidence reviewed above clearly supports the notion that there are alterations in the immune system upon examination of the brain in individuals with ASD. The strongest evidence for activation of the immune system are the studies which demonstrated histological evidence of microglia cell changes in the frontal (Vargas et al., 2005; Morgan et al., 2010, 2012a; Tetreault et al., 2012), cingulate (Vargas et al., 2005) and cerebellum (Vargas et al., 2005). Neuroimaging supports these histological findings (Suzuki et al., 2013). Evidence for disruption in immune regulation is supported by elevations in proinflammatory cytokines in brain tissue from the frontal (Li et al., 2009), cingulate (Vargas et al., 2005), and cerebellum (Wei et al., 2011) and in CSF (Vargas et al., 2005; Chez et al., 2007) derived from individuals with ASD and elevations in the expression of genes regulating proinflammatory pathways in the temporal (Garbett et al., 2008) and frontal (Young et al., 2011) areas in individuals with ASD. Although some studies have reported some negative or inconsistent results (Vargas et al., 2005; Zimmerman et al., 2005; Malik et al., 2011b), the majority of studies point to an activation of the innate immune system in the brain of individuals with ASD and some of the findings, particularly the cytokine elevations, parallel abnormal elevations in cytokines reported in non-CNS tissue in children with ASD. Although these studies suffer from small sample sizes and inconsistency in brain areas examined, together they provide support for more comprehensive research into the role of inflammation and immune dysregulation in the brain of children with ASD.

## Discussion

Although ASD is defined by observations of behaviors and is thus classified as a psychiatric disorder, recent evidence has pointed to physiological abnormalities in ASD, suggesting that ASD has a clear biological basis with features of known medical disorders. A number of studies using peripheral biomarkers have linked oxidative stress, mitochondrial dysfunction and immune dysregulation in individuals with ASD (James et al., 2009a; Mostafa et al., 2010; Zhang et al., 2010; Napoli et al., 2013; Theoharides et al., 2013; Frye et al., 2013a). Recently, studies have examined possible interactions between these abnormalities in the brain of individuals with ASD. Indeed, many of the reviewed studies have been published since 2010, including studies examining oxidative stress (8/12 studies, 67%), mitochondrial dysfunction (11/13, 85%) and immune abnormalities (8/14, 57%).

Furthermore, four studies reported that oxidative stress and mitochondrial dysfunction were linked in the brain of individuals with ASD (Palmieri et al., 2010; Chauhan et al., 2011; Rose et al., 2012b; Tang et al., 2013), whereas two studies reported a connection between oxidative stress and inflammation in the brain (López-Hurtado and Prieto, 2008; Rose et al., 2012b). One of these studies linked low GSH levels, oxidative stress, mitochondrial dysfunction and inflammation in the brain of individuals with ASD (Rose et al., 2012b). Interestingly, only one study reported no evidence of inflammation in the CSF of individuals with ASD (Zimmerman et al., 2005), whereas another study found a similar oxidative stress marker in both groups, although several findings suggestive of an adaptive response to oxidative stress were observed in the brains of individuals with autism (Muratore et al., 2013).

A recent systematic review of 112 individuals with ASD and concomitant mitochondrial disease found that only about 21% had a genetic abnormality that could account for the reported mitochondrial problem (Rossignol and Frye, 2012a). Mitochondrial dysfunction found in some individuals with autism could be related to inflammation or immune dysregulation. For example, TNF-alpha is known to inhibit mitochondrial function (Suematsu et al., 2003; Vempati et al., 2007; Samavati et al., 2008) and elevations in TNF-alpha, from individuals with ASD compared to controls, have been reported in lymphocytes (Malik et al., 2011a), amniotic fluid (Abdallah et al., 2013), CSF (Chez et al., 2007) and brain samples (Li et al., 2009). GSH protects mitochondria against the adverse effects of TNF-alpha (Fernandez-Checa et al., 1997) and GSH deficiency can lead to impaired mitochondrial function (Vali et al., 2007). Interestingly, TNF-alpha (also known as cachexin) is known to decrease mitochondrial enzymatic function, including cytochrome c oxidase (complex IV) activity (Remels et al., 2010). Some studies, including one using brain tissue, have reported complex IV overactivity in individuals with ASD rather than an inhibition of this complex (Palmieri et al., 2010; Frye and Naviaux, 2011). It may be that active inflammation results in a compensatory increase in complex IV activity so that this complex becomes overactive once the inflammation has subsided (Frye and Naviaux, 2011); such a scenario could explain why complex IV is found to be both reduced and increased in multiple sclerosis lesions depending upon whether the lesion is undergoing active inflammation or whether the inflammation has subsided (Lu et al., 2000; Mahad et al., 2009).

In addition, oxidative stress may lead to impaired mitochondrial function (Fernandez-Checa et al., 1997). For example, the mitochondrial ETC is the predominant source and major target of free radicals (Fernandez-Checa et al., 1998; Trushina and McMurray, 2007). Free radicals impair mitochondrial function (Fernandez-Checa et al., 1997; Wallace, 1999). Mitochondria are protected from oxidative stress by GSH (Fernandez-Checa et al., 1998), although other antioxidant systems are also important (such as MnSOD). However, mitochondria lack the enzymes to produce GSH and are dependent on GSH production in the cytosol (Enns, 2003; James et al., 2009a), although mitochondria do possess glutathione reductase and therefore can regenerate GSH from GSSG. Depletion of GSH can lead to mitochondrial impairment and make cells more vulnerable to damage from free radicals which originate in the mitochondria (Fernandez-Checa et al., 1997). Several studies have reported lower GSH levels (James et al., 2004, 2006, 2009b) along with a lower mitochondrial GSH reserve (James et al., 2009a) in individuals with ASD compared to controls. Oxidative stress appears to be a common feature in individuals with ASD (Chauhan and Chauhan, 2006; Frustaci et al., 2012) and may play a role in the mitochondrial dysfunction reported in some children with ASD (Frye and Rossignol, 2011; Rossignol and Frye, 2012b).

A number of studies have reported that biomarkers of oxidative stress, mitochondrial dysfunction and immune dysregulation are correlated with autism severity. While none of the studies examining brain tissue in autism correlated findings with autism severity, some studies reported that brain areas affected by oxidative stress, mitochondrial dysfunction and immune dysregulation are areas responsible for brain functions that are typically impaired in ASD (Table 4). For example, areas involved in speech processing (Sajdel-Sulkowska et al., 2011; Rose et al., 2012b), memory, social interaction, and sensory and motor coordination (Sajdel-Sulkowska et al., 2011) were reported as having oxidative stress in some studies. Therefore, some of these physiological abnormalities in the brain may account for certain symptoms of autism. It is possible that treatment of these abnormalities may lead to a reduction in autism behaviors. For example, a number of studies have reported improvements in autism using nutritional supplements and medications which can support mitochondrial function (Geier et al., 2011; Frye and Rossignol, 2012b; Rossignol and Frye, 2012b; Fahmy et al., 2013), reduce oxidative stress (Dolske et al., 1993; Chez et al., 2002; Adams and Holloway, 2004; Rossignol, 2009; Adams et al., 2011; Rossignol and Frye, 2011; Hardan et al., 2012; Ghanizadeh and Moghimi-Sarani, 2013), and decrease inflammation (Stefanatos et al., 1995; Shenoy et al., 2000; Boris et al., 2007; Bradstreet et al., 2007; Asadabadi et al., 2013; Taliou et al., 2013). Additional studies are needed to determine if these types of treatments lead to changes in oxidative stress, mitochondrial dysfunction and immune abnormalities reported in the brains of some individuals with ASD.

**Table 4:** **Regions affected along with function/findings; organized by oxidative stress, mitochondrial dysfunction and immune dysregulation**.

**Region**	**Function**	**Findings in autism compared to controls**
CSF		**Immune dysregulation:**
		• Elevated INF-gamma, MCP-1, TGF-beta2, and IL-8 (Vargas et al., 2005)
		• Elevated TNF-alpha concentration (Chez et al., 2007)
		• CSF quinolinic acid and neopterin were significantly lower and biopterin was significantly elevated (Zimmerman et al., 2005)
**FRONTAL LOBE**		
Frontal lobe	Motor control and planning, higher cognitive function, executive function (attention, working memory, planning, behavioral control), decision making, thinking	**Oxidative stress:**
		• Similar 8-hydroxyguanosine in the frontal cortex (BA 9, 22, 41, 42, or 46) between groups. Significantly lower methionine synthase mRNA along with lower levels of homocysteine and cystathionine is same areas in the autism brains, suggestive of adaptive responses to oxidative stress (Muratore et al., 2013)
		**Mitochondrial dysfunction:**
		• Elevation in lactate by MRS (Chugani et al., 1999)
		• Lower ETC complex activities (Chauhan et al., 2011) with mean reductions in ETC complexes I (31%) and V (36%) activities (Gu et al., 2013a)
		• A higher mitochondrial DNA (mtDNA) copy number (Gu et al., 2013a)
		**Immune dysregulation:**
		• Elevations in proinflammatory cytokines (including TNF-alpha, IL-6 and GM-CSF), a Th1 cytokine (IFN-gamma) and a chemokine (IL-8) (Li et al., 2009)
Middle frontal gyrus	Lexical and semantic processing, comprehension	**Immune dysregulation:**
		• Activation of microglia and astroglia (Vargas et al., 2005)
		• Anti-inflammatory cytokine tumor growth factor–1 and pro-inflammatory macrophage chemoattractant protein-1 were found to be increased (Vargas et al., 2005)
Dorsal prefrontal cortex	Working memory, planning, behavioral regulation, reasoning	**Mitochondrial dysfunction:**
		• Abnormal levels of brain markers of mitochondrial function by MRS (Minshew et al., 1993)
		**Immune dysregulation:**
		• Microglial activation (Morgan et al., 2010)
BA11 (Orbitofrontal cortex)	Emotion, reward, expectations	**Oxidative stress:**
		• Elevated 3NT levels (Sajdel-Sulkowska et al., 2011)
		**Immune dysregulation:**
		• Activation of microglia (Suzuki et al., 2013)
		• NF-KappaB expression was increased (Young et al., 2011)
BA44 (Broca's area in dominant hemisphere)	Response inhibition, music perception, speech production in the dominant hemisphere	**Oxidative stress:**
		• Increase in lipofuscin-containing cells (López-Hurtado and Prieto, 2008)
		**Immune dysregulation:**
		• Reactive gliosis (López-Hurtado and Prieto, 2008)
**PARIETAL LOBE**		
Parietal lobes		**Immune dysregulation:**
		• Activation of microglia (Suzuki et al., 2013)
BA39 (gyrus angularis)	Reading	**Oxidative stress:**
		• Increase in lipofuscin-containing cells (López-Hurtado and Prieto, 2008)
		• Elevated markers of oxidative damage (Evans et al., 2008)
		**Immune dysregulation:**
		• Reactive gliosis (López-Hurtado and Prieto, 2008)
**TEMPORAL LOBES**		
Temporal lobes		**Oxidative stress:**
		• Lowered GSH and GSH/GSSG (Chauhan et al., 2012a)
		• Higher levels of lipid hydroperoxides (Chauhan et al., 2011)
		• 8-oxo-deoxyguanosine higher (Tang et al., 2013)
		**Mitochondrial dysfunction:**
		• Lower ETC complex activities (Chauhan et al., 2011)
		**Immune dysregulation:**
		• Elevated 3-chlorotyrosine (Rose et al., 2012b)
Superior temporal gyrus	Primary auditory cortex and recognition of sound and language (in the dominant hemisphere)	**Immune dysregulation:**
		• Microglial activation (Suzuki et al., 2013)
		• Increased transcription levels of several immune-related genes (Garbett et al., 2008)
Hippocampus	Long term memory	**Oxidative stress:**
		• Elevated markers of oxidative damage (Evans et al., 2008)
BA21	Processing of sound and language (in dominant hemisphere)	**Oxidative stress:**
		• Decreased superoxide dismutase 2 activity (Tang et al., 2013)
		**Mitochondrial dysfunction:**
		• Decreased ETC complex I and IV activities (Tang et al., 2013)
		• Higher levels of mitochondrial fission proteins and lower levels of mitochondrial fusion proteins (Tang et al., 2013)
BA22 (Wernicke's area in dominant hemisphere)	Speech processing and understanding in dominant hemisphere	**Oxidative stress:**
		• Lowered GSH and GSH/GSSG (Rose et al., 2012b)
		• Increase in lipofuscin-containing cells (López-Hurtado and Prieto, 2008)
		• Elevated 3NT levels (Sajdel-Sulkowska et al., 2011; Rose et al., 2012b)
		• 3-fold higher level of oxidative damage to mitochondrial proteins (Palmieri et al., 2010)
		• Significant elevations in 8-oxo-deoxyguanosine (Rose et al., 2012b)
		**Mitochondrial dysfunction:**
		• Mean activity of the TCA cycle enzyme aconitase was significantly decreased (Rose et al., 2012b)
		**Immune dysregulation:**
		• Reactive gliosis (López-Hurtado and Prieto, 2008)
BA41/42	Auditory	**Oxidative stress:**
		• 3-fold higher level of oxidative damage to mitochondrial proteins (Palmieri et al., 2010)
**OCCIPITAL LOBE**		
BA19 (occipital lobe)	Visual feature extraction and shape recognition, visual attention	**Mitochondrial dysfunction:**
		• Decreased mitochondrial ETC complex gene expression (Ginsberg et al., 2012)
**CEREBELLUM**		
Cerebellum	Motor coordination, modulation of cognition and behavior	**Oxidative stress:**
		• Lowered GSH and GSH/GSSG (Chauhan et al., 2012a; Rose et al., 2012b)
		• Lowered glutathione peroxidase, glutathione-S-transferase, and glutamate cysteine ligase (Gu et al., 2013b)
		• Elevated markers of oxidative damage (Evans et al., 2008)
		• Higher levels of lipid hydroperoxides (Chauhan et al., 2011)
		• Elevated 3-NT (Sajdel-Sulkowska et al., 2008, 2011; Rose et al., 2012b)
		• Significant elevations in 8-oxo-deoxyguanosine (Rose et al., 2012b); trend for elevation in 8-hydroxydeoxyguanosine (Sajdel-Sulkowska et al., 2009)
		**Mitochondrial dysfunction:**
		• Lower ETC complex activities (Chauhan et al., 2011)
		• Mean activity of the TCA cycle enzyme aconitase was significantly decreased (Rose et al., 2012b)
		• Decreased mitochondrial ETC complex gene expression (Ginsberg et al., 2012)
		**Immune dysregulation:**
		• Activation of microglia (Vargas et al., 2005; Suzuki et al., 2013) and reactive astroglia (Vargas et al., 2005)
		• IL-6 was significantly increased (Wei et al., 2011)
		• Anti-inflammatory cytokine tumor growth factor–1 and pro-inflammatory macrophage chemoattractant protein-1 were found to be increased (Vargas et al., 2005)
		• Elevated 3-chlorotyrosine (Rose et al., 2012b)
**LIMBIC SYSTEM**		
Thalamus	Sensory and motor relaying and gating, cortical rhythm generator	**Mitochondrial dysfunction:**
		• Reduced expression of mitochondrial ETC genes (Anitha et al., 2013)
		• Decreased expression of mitochondrial genes (Anitha et al., 2012)
Anterior cingulate gyrus	Error detection, conflict monitoring, emotional awareness, pain	**Mitochondrial dysfunction:**
		• Reduced expression of mitochondrial ETC genes (Anitha et al., 2013)
		• Decreased expression of mitochondrial genes (Anitha et al., 2012)
		**Immune dysregulation:**
		• Activation of microglia (Vargas et al., 2005; Suzuki et al., 2013) and reactive astroglia (Vargas et al., 2005)
		• Anti-inflammatory cytokine tumor growth factor–1 and pro-inflammatory macrophage chemoattractant protein-1 were found to be increased (Vargas et al., 2005)
**BRAINSTEM**		
Pons	Autonomic function, eye movements, motor and sensory relay	**Oxidative stress:**
		• Elevated 3NT levels (Sajdel-Sulkowska et al., 2011)

## Conclusions and perspectives

Overall, the studies reviewed above provide support for the idea that oxidative stress, mitochondrial dysfunction and inflammation/immune dysfunction, which are physiological abnormalities identified in non-CNS tissue in children with ASD, are also found to affect the CNS. A few studies demonstrated the connection between these physiological abnormalities. However, there were several limitations to the studies reviewed, including small sample sizes and inconsistencies in the techniques and biomarkers studied and the brain areas examined. Because of these limitations, at this time, it is difficult to know if the findings are localized to a certain portion of the brain or whether these abnormalities are more diffuse. Another challenge is whether or not these abnormalities can be generalized to all children with ASD, or if they represent a subgroup of children with ASD. However, the consistent positive findings across studies suggest that these effects are not subtle and may be important in the pathological mechanisms that disrupt brain function in ASD.

Of interest is that many of the physiological abnormalities noted in the brain of children with ASD are also found in various other neurocognitive and psychiatric diseases. For example, studies in both Alzheimer and Parkinson disease have implicated dysfunction of ETC function (primarily complex IV in Alzheimer and primarily complex I in Parkinson) and in the dynamics of mitochondrial fission and fusion (Moran et al., 2012b), two specific mitochondrial abnormalities that have been reported in ASD. Interestingly, the mitochondria is being investigated for its role in axonal degeneration and repair in multiple sclerosis (van Horssen et al., 2012). Along with mitochondrial dysfunction, oxidative stress and inflammation have also been implicated in a wide range of neurodegenerative diseases including Alzheimer, Parkinson, multiple sclerosis, amyotrophic lateral sclerosis and Friedreich's ataxia (Calabrese et al., 2005; Nuzzo et al., 2013). Like ASD, inflammation, oxidative stress and mitochondrial dysfunction are seen in a wide range of psychiatric disorders (Dantzer et al., 2008; Ng et al., 2008; Shao et al., 2008; Burke and Miller, 2011). Thus, these pathophysiological mechanisms appear to be shared by many diseases that have cognitive and behavioral symptoms. Therefore, future research will need to investigate which pathophysiological mechanisms are shared among these diseases. Such knowledge may lead to novel treatments and strategies for preventing these pathophysiological processes, and thus neurocognitive and psychiatric diseases, from developing.

### Conflict of interest statement

The authors declare that the research was conducted in the absence of any commercial or financial relationships that could be construed as a potential conflict of interest.
